# Changements environnementaux et émergence de zoonoses

**DOI:** 10.48327/mtsi.v6i2.2026.869

**Published:** 2026-06-26

**Authors:** François MOUTOU

**Affiliations:** Société francophone de médecine tropicale et santé internationale (ancienne SPE) Institut Pasteur, 25 Rue du Dr Roux, 75015 Paris, France

**Keywords:** Changements environnementaux, Santé humaine, Sources de données, Zoonoses, Environmental Changes, Human Health, Data Sources, Zoonoses

## Abstract

L’évolution de certains paramètres planétaires (climat, écosystèmes), pressentie depuis quelques décennies et confirmée durant la seconde moitié du XX^e^ siècle, s’est nettement accélérée. La question des impacts possibles ou avérés de cette accélération est donc posée. Dans le domaine de la santé humaine, traitée ici, comme pour la santé animale et la qualité de l’environnement, des conséquences sont déjà notables. Le cas particulier des zoonoses, ces maladies infectieuses et parasitaires, transmissibles, dont les agents peuvent circuler entre humains et autres vertébrés, illustre assez bien ce à quoi ces changements peuvent conduire. Cette courte synthèse rappelle d’abord les enjeux et difficultés à surmonter pour pouvoir disposer de sources de données fiables et accessibles, puis présente quelques exemples. Une prise de conscience aux plus hauts niveaux est nécessaire pour espérer renverser la tendance.

## Abstract (en)

Changes in certain global parameters, such as climate and ecosystems, which were anticipated for several decades and confirmed during the second half of the 20^th^ century, have accelerated significantly. This raises the question of the possible or proven impacts of this acceleration. In the field of human health, as well as animal health and environmental quality, the consequences are already significant. The case of zoonoses, infectious and parasitic diseases that can be transmitted between humans and other vertebrates, illustrates where these changes may lead.

This brief overview first outlines the challenges and difficulties of ensuring access to reliable, accessible data sources and then presents a few examples. Awareness at the highest levels is necessary to reverse this trend.

**Figure:**
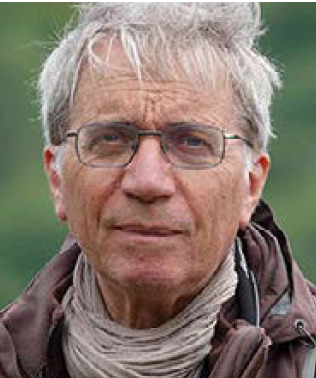

**Figure:**
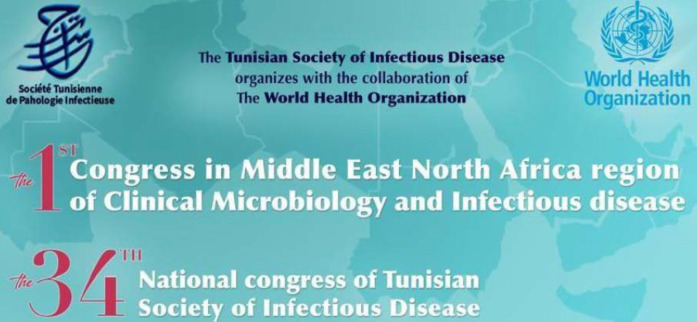

Si les activités humaines modifient les paysages et le fonctionnement des écosystèmes depuis des millénaires, l’accélération contemporaine de ces pressions environnementales est sans équivalent connu. Plusieurs facteurs peuvent l’expliquer, comme la révolution industrielle associée à l’explosion démographique depuis la fin du XVIII^e^ siècle et, surtout, le début du XIX^e^ siècle. Il s’en suit un changement rapide dans l’usage des sols, une industrialisation de l’agriculture ou encore un volume et une vitesse de déplacements et de transports de biens et de personnes à des niveaux encore jamais atteints [20]. En conséquence, on enregistre des chiffres importants pour diverses pollutions chimiques comme biologiques et une évolution climatique globale et complexe. L’impact anthropique sur les écosystèmes devient considérable (voir les rapports du Groupe d’experts intergouvernemental sur l’évolution du climat, GIEC^1^, et ceux de la Plateforme intergouvernementale scientifique et politique sur la biodiversité et les services écosystémiques, IPBES^2^). Toutes les espèces vivantes sont confrontées à ces perturbations. Cela concerne de nombreux microorganismes, leurs réservoirs, leurs vecteurs, arthropodes ou mollusques par exemple. C’est ainsi que ces grands bouleversements planétaires ont des répercussions, certaines déjà apparentes, d’autres encore potentielles, dans le domaine de la santé publique, de l’élevage et de l’agriculture, du vivant en général.

Une catégorie particulière de maladies infectieuses transmissibles rassemble les zoonoses, celles dont l’agent responsable peut circuler entre les humains et les autres vertébrés, en fait surtout les mammifères et les oiseaux. On peut y associer les maladies d’origine animale, celles dont l’agent microbien s’est adapté aux humains. Dans ce cas, après son passage initial à *Homo sapiens*, et son « humanisation », l’agent n’a plus besoin du réservoir animal pour exister. Il circule seul dans les populations humaines [29]. C’est par exemple le cas du paramyxovirus de la rougeole humaine. Issu du virus responsable de la peste bovine (maladie animale éliminée de la planète en 2011) [16], le virus ne circule plus aujourd’hui que chez les humains. La recrudescence actuelle de la rougeole n’est donc pas liée à un quelconque réservoir animal mais strictement à des comportements humains [10].

Aborder ces questions est complexe, tant les facteurs sont nombreux, de natures très diverses et s’interpénétrant régulièrement. C’est pour cela que cette synthèse ne prétend pas être exhaustive. Le sujet est trop vaste. L’enjeu serait néanmoins d’alerter sur les biais possibles dans la recherche des facteurs à l’œuvre. En effet, les données accessibles et publiées dans certaines régions, le plus souvent limitées à quelques entités morbides, ne donnent pas directement une image mondiale bien hiérarchisée des enjeux globaux. Il faut également que les méthodologies de prospection soient transparentes et accessibles. Dans un deuxième temps, quelques exemples de zoonoses seront examinés, celles pour lesquelles il semble légitime de penser que les changements environnementaux en cours interviennent déjà ou interviendront prochainement.

## Changements environnementaux, causes et conséquences

### Quelles sources de données?

La liste des changements environnementaux responsables de l’émergence potentielle de zoonoses, ou, pour celles déjà connues, de l’augmentation de leur incidence, est longue. Sans chercher l’exhaustivité, on peut citer la température dont l’augmentation moyenne mondiale est de presque 1° C du fait des émissions cumulées de gaz à effet de serre, CO_2_ entre autres, et la déforestation avec son impact sur la pluviométrie. Comme peu d’actions semblent entreprises pour y remédier, les conséquences devraient encore se poursuivre [15]. L’analyse de cette liste est complexe car une cause primaire initiale peut conduire à une conséquence, elle-même source secondaire de risques d’émergence. Le choix des sources de données est évidemment important. C’est ainsi que le Rapport annuel publié par la revue *The Lancet*, édition de 2024 [22], permet une certaine hiérarchisation et organisation dans la présentation des facteurs. C’est important pour appréhender l’impact du changement climatique, certainement le changement environnemental majeur sur la santé. Ce rapport propose cinq chapitres. Le premier (risques pour la santé, expositions et impacts), lui-même découpé en plusieurs sous-entités, est le seul à citer les maladies infectieuses en pointant quatre d’entre elles, à savoir la dengue, la malaria, *Vibrio* (agent du choléra) et la maladie liée au virus West Nile. Deux sont des zoonoses. Les quatre autres chapitres concernent l’adaptation (planification et résilience), l’atténuation (et les co-bénéfices pour la santé), l’économie (et les finances) et enfin l’engagement public et politique pour la santé face au changement climatique. Clairement, l’émergence de maladies, dont les zoonoses, ne représente qu’un seul des nombreux enjeux associés à l’impact des changements environnementaux sur la santé.

L’étape suivante consiste à analyser les sources de données directement liées aux maladies. Cela permet de vérifier, *a priori*, quels sont les sujets (agents pathogènes, hôtes, vecteurs) qui ont fait l’objet de recherches, dans quels pays et selon quels protocoles. La synthèse de Van de Vuurst et Escobar [28] énumère et hiérarchise les pays les plus pris en compte, les agents pathogènes les plus étudiés, les espèces vectrices et réservoirs les plus examinés, les pathologies les plus couvertes. Ceci illustre le fait que tous ne sont pas abordés ni traités de la même manière, ce qui peut déboucher sur un certain déséquilibre de traitement, voire un certain nombre de biais possibles en cas de comparaisons. Les cartes publiées dans l’article illustrent bien les zones encore très peu étudiées, les microorganismes et les vecteurs bien ou moins bien suivis et donc les inégalités d’informations et de traitements qui en résultent. Il reste des efforts à fournir pour assurer à de nombreuses populations humaines et à beaucoup d’écosystèmes, les mesures d’anticipation possibles et légitimes face aux risques auxquels ils pourraient être exposés. La mondialisation déplace aisément les lieux d’émergence des facteurs de changement vers des lieux d’apparition et d’exposition à leurs conséquences parfois bien éloignés de la source véritable.

### Quels changements environnementaux?

Selon les dernières projections du GIEC^1^ et de l’IPBES^2^, dans l’hypothèse d’un réchauffement planétaire de 2 °C, pas moins de 18 % des insectes, 16 % des plantes et 8 % des vertébrés devraient perdre plus de la moitié de leur aire d’extension géographique et 5 % des espèces seraient estimées en voie d’extinction. Les effets directs sur la santé humaine par simple immobilité physique à cause de la chaleur excessive seraient déjà élevés^3^.

Dans la liste des autres facteurs majeurs, l’évolution de la démographie humaine apparaît assez vite, à la fois en chiffres absolus (plus de huit milliards d’humains sur Terre aujourd’hui), en rapidité de croissance (à peine deux siècles pour passer de un à huit milliards pour une espèce apparue il y a environ 300 000 ans) et en répartition (certaines mégalopoles dépassent 20 millions d’habitants, dans des conditions souvent critiques) [21]. Le changement d’usage des sols (déforestation, urbanisation mal encadrée, industrialisation, imperméabilisation des surfaces, agriculture intensive, gestion de l’eau), peut conduire à de nombreuses pollutions, possiblement néfastes pour les systèmes immunitaires des individus exposés^4^.

L’accroissement et la vitesse du transport (humains, autres vivants, biens) augmentent la probabilité de circulation des agents et vecteurs, connus ou moins connus, de nombreuses maladies. Le premier voyage transatlantique de Christophe Colomb avait duré plus de deux mois, dont encore 35 jours après l’escale aux Canaries^5^. Aujourd’hui un vol régulier Paris - New York prend 6 heures, moins que la durée d’incubation de pratiquement toutes les maladies infectieuses connues.

À côté de la démographie humaine, on peut citer l’évolution de celle des autres mammifères (espèces domestiques et sauvages) et des oiseaux (domestiques et sauvages) du début du néolithique à aujourd’hui. Dans ce cas l’estimation se fonde sur la biomasse, sujet exploré ces dernières années [1,7,23,25]. C’est ainsi que l’on peut supposer qu’il y a 10 000 ans, la biomasse des mammifères sauvages terrestres représentait 99 % du total et les humains le 1 % restant. Il n’y avait alors pas de mammifères domestiques, en négligeant les premiers chiens. Aujourd’hui, les estimations évaluent la masse des mammifères domestiques, surtout des ruminants, entre 60 et 67 % du total. Les humains représenteraient 32 à 36 % de l’ensemble et le reste (1 à 4 %) correspondrait aux mammifères sauvages terrestres (environ 6 500 espèces). Chez les oiseaux, il n’y avait pas non plus de formes domestiques il y a 10 000 ans. Aujourd’hui, le rapport serait d’environ 30 % pour les sauvages (10 000 espèces) et 70 % pour les domestiques. Le grand nombre d’animaux domestiques, représentés par un petit nombre d’espèces (une vingtaine d’espèces significatives chez les mammifères comme chez les oiseaux), pourrait représenter un risque particulier. D’autant plus qu’ils sont régulièrement élevés en grands effectifs, de races ou de lignées génétiques assez homogènes, sous forme de lots du même âge, dans des élevages eux-mêmes géographiquement concentrés et régulièrement proches des humains. Cette situation crée également toute une série de situations où faunes sauvage et domestique se côtoient, sachant que les formes sauvages de certaines espèces domestiques existent toujours, ce qui peut faciliter la circulation d’agents zoonotiques, dans les deux sens, entre réservoir sauvage et populations humaines via les troupeaux domestiques mais aussi entre troupeaux domestiques et populations sauvages. Ces relations entre la faune sauvage, pourtant en régression, et la faune domestique, plutôt en expansion, représentent un important défi.

### Animaux sauvages - animaux domestiques

Dans de nombreuses régions de la planète, les troupeaux d’herbivores domestiques et sauvages se côtoient sur les mêmes pâturages. Cela pose la question du partage de l’espace, à la fois en termes d’accès à la ressource alimentaire et de risque de transmission de maladies.

Depuis 2011, un cas assez emblématique de la complexité d’approche et de gestion de certaines situations existe dans les Alpes françaises, plus précisément dans le massif du Bargy, en Haute-Savoie [9]. Deux cas de brucellose humaine à *Brucella melitensis* sont diagnostiqués fin 2011, sans pouvoir alors être expliqués. Pour rappel, le pays est officiellement indemne de brucellose depuis la fin du XX^e^ siècle. Il n’existe pas de réservoir sauvage connu. Puis un cas bovin est identifié dans la même vallée début 2012, avant que des cas ne soient suspectés puis confirmés à l’automne 2012 chez des ongulés sauvages, essentiellement des bouquetins (*Capra ibex*), espèce protégée, donc non chassée. Des ruminants domestiques, moutons ou chèvres, ont pu contaminer les bouquetins dans les années 1980-1990, avant l’élimination, locale et nationale, de la bactérie. Une dizaine d’années plus tard, la bactérie revient dans un troupeau bovin puis passe à deux humains *via* des fromages au lait cru. Cet épisode associe plusieurs facteurs. La présence trop régulière de chèvres domestiques au contact des bouquetins interroge sur la gestion des troupeaux en estive. Le réchauffement climatique favorise le maintien en haute montagne d’animaux domestiques, pourtant peu ou mal adaptés à ces milieux. La bactérie a circulé dans la faune sauvage pendant environ une décennie avant sa transmission vers les bovins mais les contacts bouquetins - bovins ne semblent pas connus. Il pourrait exister un facteur non identifié reliant les deux espèces. La gestion de cet épisode avec l’abattage de plusieurs centaines de bouquetins a posé plus de problèmes qu’elle n’en a résolus. En 2025, le foyer « sauvage » existe toujours mais il n’y a pas eu de nouveau cas humain depuis ceux de 2011.

À une tout autre échelle, la situation de la rage dans le monde représente un grand enjeu de santé publique. L’OMS estime entre 50 000 et 60 000 le nombre de morts humaines par an. Un programme dédié annonce son éradication pour 2030^6^. Maladie largement répartie sur la planète, la rage peut être transmise par un grand nombre de mammifères sauvages et domestiques. Les réservoirs de virus varient selon les régions, les continents et les environnements. Pourtant, les données disponibles montrent que l’essentiel, si ce n’est la quasi-totalité des cas humains, sont consécutifs à des morsures de chiens domestiques, souvent errants. La population mondiale de chiens domestiques est estimée à 700 millions d’individus par l’Organisation mondiale de la santé animale (OMSA), dont malheureusement une grande partie ne serait pas sous le contrôle de propriétaires^7^. Or le changement climatique, souvent orienté vers un réchauffement, associé à la mauvaise gestion des déchets organiques (élevages, abattoirs, équarrissages, restes de nourriture) favorise la croissance des populations canines hors du contrôle des humains. La réponse est pourtant connue. Elle consisterait à les contrôler (reproduction en particulier) et à les vacciner. Dans les faits, les services de santé des pays les plus touchés ne s’occupent que des humains. Leurs services vétérinaires priorisent les animaux d’élevage et de rente, pas ceux de compagnie. La mauvaise gestion humaine de la croissance de ces populations canines, malgré les nuisances qu’elles entraînent, conduisent à une dégradation de la santé globale. Au-delà des humains, les chiens contaminent de nombreuses autres espèces de mammifères, certaines menacées [8,27].

Le dernier exemple associant de près faune domestique, faune sauvage et humains concerne la grippe aviaire [19]. Depuis le début du XXI^e^ siècle, quelques influenzavirus A font parler d’eux, en particulier H5N1, même s’il n’est pas le seul. Les premières émergences en Europe dans les années 2005-2006 étaient limitées par rapport à ce qui est apparu une quinzaine d’années plus tard. Le virus circule bien avec des oiseaux migrateurs sauvages entre zones de reproduction, souvent loin des zones habitées par les humains et les zones d’hivernage, souvent plus proches de régions agricoles. Pour autant, les voies de contamination des élevages ne sont pas toutes connues. En effet, les grands migrateurs sont rarement vus au contact des élevages industriels. Un ou des relais locaux doivent exister mais leur nature et leurs identités ne sont pas encore connues. Il est également apparu que de grands élevages densément peuplés et en grande concentration dans un même bassin de production de volailles pouvaient se contaminer les uns les autres par voie aérienne, simplement par proximité. L’arrivée d’une souche virale peut être associée à des oiseaux sauvages, mais le développement d’épizooties locales semble plus lié aux élevages eux-mêmes et à leurs pratiques. Certains virus qui circulent dans les espèces sauvages ne sont jamais retrouvés en élevages. Même si les voies de migration des oiseaux sauvages évoluent dans le temps, les méthodes d’élevage en milieu avicole (déplacements et regroupements des lots durant la croissance) peuvent aussi contribuer à diffuser les virus. Là encore, plusieurs facteurs assez différents, humains et environnementaux, interviennent. La réponse actuelle qui consiste à vacciner certains oiseaux d’élevage ne prend peutêtre pas tous les éléments du problème en compte, par exemple la concentration et la densité des élevages. La découverte du passage d’une lignée du virus H5N1 dans les grands élevages laitiers des USA au printemps 2024 ouvre une possible nouvelle étape de cette épizootie. Ce virus adapté aux bovins circule par le lait et, plusieurs cas humains ont déjà été diagnostiqués. À ce jour, les cas humains ne transmettent pas le virus. La question du risque d’émergence d’une souche mieux adaptée à la transmission interhumaine est néanmoins posée à un moment où les USA semblent moins suivre leur situation sanitaire nationale, humaine et animale. Comme bien montré par Peacock *et al*. [19], depuis les années 1990 le nombre de passages annuels de virus influenza, en particulier certaines souches de H5N1, vers des mammifères ne fait que croître. Ceci augmente d’autant le risque d’une adaptation aux humains d’une de ces souches.

## Climat et déforestation

### Virus Hendra

Le virus Hendra est un paramyxovirus du même genre que le virus Nipah, infectant chevaux et humains, responsable chez les chevaux d’une pneumonie potentiellement mortelle. Découvert dans les années 1990 en Australie, il a récemment fait l’objet d’une étude sur 25 ans pour essayer de comprendre son écologie, son épidémiologie et les facteurs expliquant son émergence irrégulière. Le nombre de cas humains est resté faible à ce jour mais le taux de létalité justifie d’accroître les connaissances sur les risques associés. L’étude d’Eby *et al*. [4] associe cette émergence à des facteurs climatiques et à des facteurs environnementaux, de plus en plus corrélés à des paramètres humains. Se combinent la déforestation qui a suivi l’arrivée des Européens sur la côte est de l’Australie, la fragmentation régulière des massifs forestiers encore présents, ainsi que le rythme de floraison des essences locales, en particulier celles à floraison hivernale, dépendantes des variations apportées par l’oscillation australe (ENSO, El Niño). À partir de ces mêmes éléments, l’étude de Becker *et al*. [2] propose des critères permettant d’anticiper les années à risque d’émergence du virus. Le réservoir naturel du virus Hendra est la roussette à tête grise (*Pteropus poliocephalus*), grande chauve-souris frugivore australienne. La régression des forêts naturelles et les déficits de ressources alimentaires (fleurs, fruits) certains hivers, certaines années, la contraignent à de plus grands déplacements, y compris jusque dans les vergers plantés par les arboriculteurs, ce qui les rapproche des zones habitées. Le système immunitaire des chauves-souris maîtrise moins bien la multiplication virale en cas de stress physiologique à la suite d’une recherche accrue de nourriture. Les animaux peuvent alors excréter plus facilement le paramyxovirus. Le passage vers les humains se fait *via* la contamination de chevaux, exposés dans leurs paddocks, quand ceux-ci sont plantés d’arbres fruitiers qui attirent les roussettes. Les chevaux se contaminent probablement en consommant des débris végétaux perdus par les roussettes, comme des morceaux de fruits contaminés par leur salive, ou du fourrage souillé par leurs déjections. Curieusement, alors que l’Australie est habitée par les humains depuis plusieurs dizaines de millénaires, les communautés aborigènes actuelles ne semblent pas connaître le virus, y compris celles chez lesquelles la chasse et la consommation des roussettes étaient possibles et pratiquées [14]. Dans ce cas de figure, l’existence du virus semble bien antérieure à l’arrivée des Européens, mais des modifications environnementales récentes, depuis seulement deux siècles, expliqueraient cette émergence. Cela combine la dégradation de l’habitat du réservoir, une action indirecte sur le climat *via* El Niño, la plantation de vergers proches des zones habitées et l’introduction d’un mammifère placentaire domestique, le cheval, capable de transférer le virus depuis le réservoir animal sauvage jusqu’aux humains. Aujourd’hui, le risque pour un humain de rencontrer le virus est plus élevé en zone agricole que dans les forêts les moins perturbées [4].

### Syndrome respiratoire aigu sévère (SRAS-1 et SRAS-2)

Les leçons que l’on pourrait tirer de la compréhension des deux pandémies de SRAS (SRAS-1 en 2002-2003 et SRAS-2 ou Covid-19 depuis fin 2019) ne sont pas encore toutes écrites [18]. D’une part, l’histoire et les données épidémiologiques associées sont certainement complexes, mais de l’autre – et dans les deux cas –, le terrain des émergences se situe en Chine et les enquêtes et recherches internationales qui auraient pu avoir lieu ne se sont pas encore concrétisées fin 2025. Les deux virus en cause sont des coronavirus, probablement portés par des chiroptères. Pourtant à ce jour, les deux virus responsables n’ont pas été identifiés chez une chauve-souris et la question du passage aux humains reste toujours ouverte. En 2002-2003, un petit carnivore asiatique de la famille des viverridés, la civette palmiste masquée (*Paguma larvata*), récemment élevée en captivité dans le sud de la Chine, a peut-être joué le rôle de lien entre les chauves-souris et les humains. Pour la Covid-19 il n’y a pas encore de réponse à la question. La tradition des marchés « humides » (*wet markets*) où de nombreux animaux de diverses espèces se côtoient dans de mauvaises conditions d’hygiène a été pointée en 2002-2003. Les autorités chinoises avaient alors annoncé la fin de ces pratiques hautement à risque. Il est pourtant difficile de les exclure en 2019 lors du démarrage de la Covid-19, mais aucune image n’a circulé fin 2019, début 2020, illustrant par exemple la réalité du marché de Wuhan incriminé, avant le début de l’émergence de la Covid-19. Seul le marché vide et nettoyé a été montré, *a posteriori*.

Dans les deux cas, les virus sont bien d’origine animale mais après leur adaptation aux humains, ils ont été capables d’entraîner deux pandémies qui se sont développées sans nécessiter le moindre retour vers le réservoir animal. Les hypothèses cherchant à expliquer l’origine de la pandémie de Covid-19 combinent : travail dans des mines du sud de la Chine où gîtaient des chauves-souris rhinolophes (*Rhinolophus* spp.), des élevages d’animaux à fourrure comme des chiens viverrins (*Nyctereutes procyonides*), une fuite/accident de laboratoire, le tout dans un environnement humain, social, environnemental et économique, en forte transformation.

Dans le cas du virus SRAS-CoV-2, ce sont les humains qui ont contaminé toute une série de mammifères domestiques (chiens, chats, visons d’élevage), de compagnie (hamsters) ou même sauvages (cerf de Virginie aux USA) [17].

### Zoonoses transmises par les rongeurs

Pour certaines maladies zoonotiques déjà existantes, le risque est plus direct et mieux connu et les prévisions reposent sur des éléments plus tangibles. C’est le cas des maladies bactériennes et virales dont les agents responsables sont portés par diverses espèces de rongeurs, champêtres ou volontiers commensaux. Plusieurs schémas épidémiologiques sont possibles avec ces espèces (plus de 2 500 connues à ce jour).

Les modifications du rythme des précipitations entre les saisons sèches et les saisons des pluies en Afrique de l’Ouest pourraient conduire à une augmentation de l’incidence de la maladie de Lassa (*Mammarenavirus*, arénaviridae) au Nigeria par exemple^8^. C’est ce que les autorités locales anticipent. Les rongeurs responsables sont des rats à mamelles multiples (*Mastomys* spp*.*), volontiers commensaux. Il est intéressant de noter une récente étude qui semble suggérer que l’envahissement actuel de certaines régions de l’Afrique de l’Ouest par le rat noir (*Rattus rattus*), introduit en Afrique, concurrent écologique asiatique des rats à mamelles multiples mais mauvais réservoir du virus, aurait un effet bénéfique sur le risque humain de fièvre de Lassa [5].

Les Hantavirus des campagnols roussâtres (*Clethrionomys* spp*.)* européens pourraient bénéficier de l’extension actuelle des surfaces boisées et des activités de plein air. Dans le cas des rongeurs volontiers amphibies, ce sont les leptospiroses et les amibes (*Giardia* spp.) qui pourraient profiter d’une plus grande proximité baigneurs / rongeurs autour des derniers plans d’eau accessibles en période de sécheresse. Plus que l’émergence de nouveaux risques c’est plutôt l’évolution des risques actuels qu’il faudrait savoir prévoir (lesquels) et anticiper (de quelles manières) [6]. Le communiqué du 15 juillet 2022 de l’Académie nationale de médecine^9^ dresse une liste des dangers potentiels.

## Zoonoses vectorisées, insectes, tiques

Le champ des maladies vectorisées est immense et il est vrai que cela concernait surtout des pays de la zone intertropicale et de ses marges jusqu’à la fin du XX^e^ siècle. Le passage au XXI^e^ siècle annonce clairement que la zone d’influence de ces entités morbides s’agrandit. La région méditerranéenne et les pays de climat tempéré proches sont maintenant concernés.

Un exemple bien étudié est celui des leishmanioses, le long des rivages de la Méditerranée [12] et en Amérique du Sud. Selon les pays, le détail des données épidémiologiques diffère mais on retrouve les mêmes catégories d’acteurs. Différentes espèces de phlébotomes vecteurs vont se nourrir sur différents hôtes. Les réservoirs sauvages existent toujours mais l’intervention régulière du chien domestique, toujours proche des humains, illustre le rôle complexe de notre espèce. Au centre de l’Espagne, la leishmaniose à *Leishmania infantum* remonte vers le nord, aidée par les chiens domestiques et les populations de lagomorphes, lièvre ibérique (*Lepus granatensis*) et lapin de garenne (*Oryctolagus cuniculus*), soutenues et entretenues par les activités cynégétiques [3]. Dans le sud de la France, c’est l’évolution de villages anciennement ruraux en zones d’habitat résidentiel autour des grandes agglomérations qui a profité aux vecteurs. Les chiens domestiques de compagnie sont d’autant plus présents [12]. En Amérique du Sud une étude a ciblé l’« arc de déforestation amazonien » dans l’état du Mato Grosso brésilien [13]. La liste des vecteurs et des réservoirs potentiels vis à vis des leishmanies locales (*Leishmania* spp.) est longue mais deux espèces importantes émergent, le tatou à neuf bandes (*Dasypus novemcinctus*) et le chien domestique. Dans ce cas, ce sont bien les humains qui vont au-devant des parasites et des vecteurs. Le tatou supporte les changements et survit dans les habitats transformés tandis que le chien suit les humains. À nouveau, il s’agit de maladies déjà bien connues mais, localement, leur maintien et leur propagation combinent et cumulent divers facteurs dont certains parfaitement anthropiques. De plus, le défrichement d’espaces naturels encore non ou peu habités peut aboutir à de vraies émergences (agent pathogène encore inconnu) mais il ne s’agit pas toujours du schéma le plus fréquent. À côté du suivi de l’extension géographique de maladies vectorielles, il est important d’anticiper diverses émergences possibles en développant et renforçant des réseaux de surveillance des vecteurs eux-mêmes. L’entomologie médicale et vétérinaire, trop longtemps oubliée, voire ignorée, doit être remise en avant. Le cas d’*Aedes albopictus* en est très représentatif. L’envahissement contemporain de l’Europe est spectaculaire, encore faut-il les compétences et les outils pour le démontrer et l’analyser. L’espèce était seulement présente sur la bordure méditerranéenne française en 2014. Elle est maintenant identifiée dans les 13 régions de l’Hexagone. Elle manque seulement dans quelques départements du nord-ouest et du nord-est^10^. Les plus de 750 cas autochtones de chikungunya recensés durant l’été 2025 représentent un record absolu pour le pays. Les espaces ultramarins, non abordés ici, rencontrent des situations dont la France hexagonale finit par se rapprocher.

Il faut encore des compétences pour suivre et identifier les moustiques, mais aussi les culicoïdes, les phlébotomes, les simulies, voire les réduves pour les insectes. Il ne faut pas oublier les acariens. Les maladies transmises par les tiques évoluent également [11]. L’identification récente et sans doute l’installation de la tique *Hyalomma marginatum* dans le sud de la France, réservoir et vecteur connu de la fièvre hémorragique de Crimée Congo, ne peut qu’inquiéter [26]. Un travail collaboratif entre médecins, épidémiologistes, vétérinaires, entomologistes, écologues, climatologues, élus et citoyens est nécessaire.

## Conclusion

Aborder la question des liens possibles entre changements environnementaux et émergences de zoonoses pose de nombreuses questions de méthodes. La liste des exemples ici proposés ambitionne essentiellement de montrer la diversité des cas de figure et l’intrication régulière de plusieurs facteurs explicatifs dont de nombreux paramètres anthropiques. Il existe d’autres manières d’aborder la question, comme celle, beaucoup plus structurée, proposée par Rupasinghe *et al*. [24]. Leur schéma final combine pas moins de 13 entités morbides à caractère zoonotique avec 8 modèles de transmission possibles, 5 vectoriels plus les voies alimentaire, aquatique et aérienne. Ceci permet de rappeler deux notions importantes mais distinctes que sont le danger et le risque. L’agent de la rage canine, celui de la peste bubonique, ou celui de la fièvre hémorragique à syndrome rénal, sont tous des dangers. Mais, le risque qui en découle, la probabilité d’être mordu par un chien enragé, piqué par une puce de rat noir porteuse de *Yersina pestis*, ou d’inhaler un aérosol contenant le virus Puumala, dépend bien souvent de comportements conduisant certains humains à s’exposer à ces dangers. Le cheminement du danger au malade peut emprunter une multitude de voies. Une bonne information, une bonne prévention, permettent de contrôler et de maîtriser en amont les risques, sans nécessairement chercher systématiquement à éliminer le danger, c’est à dire souvent son réservoir, voire son écosystème. Enfin, une prise de conscience à tous les niveaux est nécessaire, depuis les individus jusqu’aux gouvernants.

## Remerciements

Pour leur invitation, leur accueil et leur disponibilité, je souhaite remercier très sincèrement les organisateurs de ce beau colloque, riches de présentations de qualité et de rencontres appréciées. Je remercie également mes collègues de la SFMTSI pour leur soutien et leurs remarques constructives.

## Financement

Aucun.

## Déclaration de liens d’intérêt

Aucun lien d’intérêt n’a été déclaré.

## Footnotes

1 https://www.ecologie.gouv.fr/actualites/publication-du-6e-rapport-synthese-du-giec

2 https://www.ipbes.net/global-assessment

3 https://www.thelancet.com/journals/langlo/article/PIIS2214-109X(25)00518-2/fulltext

4 GEO-7 https://www.unep.org/news-and-stories/press-release/ un-report-investing-planetary-health-would-deliver-higher-gdp-fewer

5 https://www.assistancescolaire.com/eleve/5e/histoire/reviser-une-notion/5hdm03

6 https://rabiesalliance.org/

7 https://www.woah.org/fr/article/stepping-up-dog-population-management-to-achieve-rabies-elimination/

8 ProMED, 29 décembre 2024

9 https://www.academie-medecine.fr/entre-le-bien-etre-du-rat-degout-et-la-sante-publique-faut-il-choisir/

10 https://sante.gouv.fr
