# Supplementary material for: Environmental changes and the emergence of zoonoses
Source: Med Trop Sante Int. 2026 Jun 26;6(2):mtsi.v6i2.2026.869. [Article in French] doi: 10.48327/mtsi.v6i2.2026.869 (PMC13423293; doi:10.48327/mtsi.v6i2.2026.869)
Supplement: Related texts

# Research article: Environmental changes and the emergence of zoonoses

Although human activities have altered landscapes and ecosystem function for millennia, the acceleration of these environmental pressures in the contemporary era is unprecedented. Several factors may explain this phenomenon, including the Industrial Revolution and the population explosion that began in the late 18^th^ and early 19^th^ centuries. These factors have led to rapid changes in land use, the industrialization of agriculture, and unprecedented levels of travel and transportation of goods and people in terms of both volume and speed [20]. Consequently, significant levels of chemical and biological pollution, as well as complex global climate change, have emerged. The impact of humans on ecosystems has become considerable (see the reports of the Intergovernmental Panel on Climate Change [IPCC^1^] and the Intergovernmental Science-Policy Platform on Biodiversity and Ecosystem Services [IPBES^2^]). All living species face these disruptions. This includes numerous microorganisms, their reservoirs, and their vectors, such as arthropods and mollusks. Thus, these major global upheavals have repercussions—some already apparent and others potential—in public health, livestock farming, agriculture, and life in general.

One category of communicable infectious diseases includes zoonoses, which are diseases that can circulate between humans and other vertebrates, primarily mammals and birds. This category also includes diseases of animal origin, in which the microbial agent has adapted to humans. After its initial transmission to *Homo sapiens* and its “humanization,” the agent no longer requires an animal reservoir to survive. It circulates independently within human populations [29]. One example is the paramyxovirus that causes human measles. Derived from the virus responsible for rinderpest, an animal disease that was eradicated in 2011 [16], the measles virus now circulates exclusively among humans. Therefore, the current resurgence of measles is strictly linked to human behaviors, not to any animal reservoir [10].

Given that the factors are numerous, highly diverse, and frequently intertwined, addressing these issues is complex. For this reason, this overview is not intended to be exhaustive. The subject is too broad. However, the aim is to draw attention to potential biases in identifying the factors at play. The data available and published in certain regions, which are often limited to a few disease entities, do not directly provide a well-structured global picture of the overarching issues. Research methodologies must also be transparent and accessible. Next, we will examine a few examples of zoonoses for which it is reasonable to assume that ongoing environmental changes are already having an impact, or will in the near future.

## Environmental changes: causes and consequences

### What data sources?

The list of environmental changes that could lead to the emergence of new zoonoses, or increase the incidence of known ones, is long. Without claiming to be exhaustive, we can cite temperature, which has risen by an average of nearly 1°C globally due to cumulative greenhouse gas emissions (including CO₂), and deforestation, which impacts rainfall. Since few measures appear to be underway to address these issues, the consequences are likely to continue [15]. Analyzing this list is complex because an initial primary cause can lead to a consequence that itself becomes a secondary source of emerging risks. The choice of data sources is important. The Annual Report published by *The Lancet* in 2024 [22] provides a certain hierarchy and structure in presenting these factors. This hierarchy and structure are important for understanding the impact of climate change, undoubtedly the most significant environmental change affecting health. The report consists of five chapters. The first chapter, “Health Risks, Exposures, and Impacts,” is divided into several subsections and is the only one to mention infectious diseases. It highlights four of them: dengue, malaria, *Vibrio* (the causative agent of cholera), and West Nile virus disease. Two of these are zoonoses. The other four chapters address adaptation planning and resilience, mitigation and co-benefits for health, the economy and finance, and public and political commitment to health in the face of climate change. Clearly, the emergence of diseases, including zoonoses, is just one of the many challenges associated with environmental changes impacting health.

The next step is to analyze data sources directly related to diseases. This allows us to verify *a priori* which topics (pathogens, hosts, and vectors) have been studied, in which countries, and according to which protocols. Van de Vuurst and Escobar’s review [28] lists and ranks the countries most frequently studied, the most commonly investigated pathogens, the most examined vector and reservoir species, and the most extensively covered diseases. This illustrates that these are not all addressed or treated in the same way, which can lead to imbalances in coverage and potential biases when making comparisons. The maps published in the article clearly illustrate the understudied areas, well- or poorly-monitored microorganisms and vectors, and the resulting inequalities in information and treatment. Further efforts are needed to ensure that numerous human populations and ecosystems have access to legitimate preventive measures to address potential risks. Globalization easily shifts the locations where drivers of change emerge to places where their consequences manifest and where people are exposed to them. These locations are sometimes far removed from the actual source.

### What environmental changes?

According to the latest projections from the IPCC¹ and IPBES², assuming a 2°C increase in global temperature, at least 18% of insects, 16% of plants, and 8% of vertebrates are expected to lose more than half of their geographic range. Additionally, 5% of species are estimated to be at risk of extinction. The direct effects on human health due to physical immobility caused by excessive heat are already significant^3^.

Among the other major factors, changes in human demographics are quite striking, including the absolute number of people on Earth (more than 8 billion today), the growth rate (it took barely two centuries to go from 1 to 8 billion for a species that appeared about 300,000 years ago), and the distribution (some megacities have more than 20 million inhabitants who often live in critical conditions) [21]. Changes in land use, such as deforestation, poorly regulated urbanization, industrialization, soil sealing, intensive agriculture, and water management, can lead to various forms of pollution that may harm the immune systems of exposed individuals^4^.

The growth and speed of transportation (of people, living beings, and goods) increase the likelihood of spreading known and lesser-known disease agents and vectors. Christopher Columbus’s first transatlantic voyage took more than two months, including 35 days after stopping in the Canary Islands^5^. Today, a scheduled flight from Paris to New York takes six hours—less than the incubation period for virtually all known infectious diseases.

In addition to human demographics, we can examine demographic trends of other mammals and birds, both domestic and wild, from the early Neolithic period to the present. In this case, the estimate is based on biomass, a topic that has been explored in recent years [1,7,23,25]. Thus, we can assume that, 10,000 years ago, wild terrestrial mammals accounted for 99% of the total biomass, while humans accounted for the remaining 1%. At that time, there were no domesticated mammals apart from the earliest dogs. Today, estimates place the mass of domestic mammals, primarily ruminants, at 60 to 67 percent of the total. Humans are estimated to account for 32 to 36 percent of the total, and the remaining one to four percent consists of wild terrestrial mammals (approximately 6,500 species). Similarly, there were no domesticated bird species 10,000 years ago. Today, the ratio is estimated to be 30% wild species (approximately 10,000 species) and 70% domesticated species. The large number of domesticated animals, represented by a small number of species (about twenty significant species among mammals and birds), could pose a particular risk. This is especially true since they are regularly raised in large, homogeneous herds, of the same genetic lineage, in groups of the same age, on farms that are geographically concentrated and often located in close proximity to humans. This situation creates many scenarios in which wild and domestic animals come into close contact. Wild forms of certain domestic species still exist, which facilitates the spread of zoonotic agents between wild reservoirs and human populations via domestic herds and between domestic herds and wild populations. The interactions between declining wildlife and expanding domesticated animals present a significant challenge.

### Wild animals - Domestic animals

In many regions of the world, herds of domestic and wild herbivores share the same pastures. This raises issues regarding resource sharing, including access to food and the risk of disease transmission.

Since 2011, the French Alps have been home to an emblematic case of the complexity of addressing and managing such situations, specifically in the Bargy Massif in Haute-Savoie [9]. In late 2011, two cases of human brucellosis caused by *Brucella melitensis* were diagnosed, though the cause could not be determined. It should be noted that the country has been officially brucellosis-free since the end of the 20^th^ century. There is no known wild reservoir. Then, in early 2012, a case of brucellosis in cattle was identified in the same valley. Later, in fall 2012, suspected cases were confirmed among wild ungulates, primarily ibex (*Capra ibex*), a protected species that is not hunted. Domestic ruminants, such as sheep or goats, may have infected the ibex in the 1980s and 1990s, before the bacterium was eradicated at the local and national levels. About a decade later, the bacterium reappeared in a cattle herd and spread to two humans *via* raw-milk cheeses. This episode involved several factors. The frequent contact between domestic goats and ibex during the summer grazing season raises questions about herd management. Global warming is enabling domestic animals to remain in high-mountain areas despite their poor adaptation to these environments. The bacterium circulated among wildlife for about a decade before being transmitted to cattle. However, there appear to be no known contacts between ibex and cattle. There may be an unidentified factor linking the two species. The response to this outbreak, which involved culling several hundred ibex, created more problems than it solved. As of 2025, the “wild” outbreak persists, though there have been no new human cases since 2011.

On a different scale entirely, the global rabies situation poses a significant public health challenge. The WHO estimates that rabies causes between 50,000 and 60,000 human deaths per year. A dedicated program aims to eradicate the disease by 2030^6^. Widespread across the globe, rabies can be transmitted by many wild and domestic mammals. Virus reservoirs vary by region, continent, and environment. However, available data show that most human cases result from bites by domestic dogs, often strays. The World Organization for Animal Health (WOAH) estimates the global population of domestic dogs at 700 million, a large proportion of which are unfortunately not under the control of owners^7^. Climate change, often characterized by warming, combined with poor management of organic waste from livestock farms, slaughterhouses, rendering plants, and food scraps, promotes the growth of uncontrollable canine populations. The solution is well known. It involves controlling their numbers, particularly through birth control, and vaccinating them. In reality, health services in the most affected countries focus solely on humans. Their veterinary services prioritize livestock and farm animals over companion animals. Human mismanagement of these growing canine populations—despite the nuisances they cause—leads to a deterioration in overall health. Beyond humans, dogs infect many other mammalian species, some of which are endangered [8,27].

The most recent example of the close link between domestic animals, wildlife, and humans involves avian influenza [19]. Since the beginning of the 21^st^ century, several influenza A viruses have garnered attention, particularly H5N1, though it is not the only one. The initial outbreaks in Europe in 2005–2006 were limited compared to the outbreaks that emerged about fifteen years later. The virus spreads readily via wild migratory birds between their breeding grounds, which are often far from human settlements, and their wintering grounds, which are often closer to agricultural regions. However, not all transmission routes to poultry farms are known. In fact, long-distance migrants rarely come into contact with industrial poultry farms. One or more local intermediate hosts must exist, but their identities and nature are unknown. It has also become apparent that large, densely populated poultry farms concentrated within the same production region can transmit the virus to one another through the air simply due to their proximity. While the arrival of a viral strain may be associated with wild birds, the development of local epizootics appears to be more closely linked to the farms themselves and their practices. Some viruses found in wild species are never found on farms. Although the migration routes of wild birds change over time, poultry farming practices, such as moving and grouping flocks during the rearing period, can contribute to the spread of viruses. Once again, several different factors—both human and environmental—come into play. The current response, which involves vaccinating certain farmed birds, may not address all aspects of the problem, such as the concentration and density of poultry farms. In the spring of 2024, the discovery of a strain of the H5N1 virus circulating in large dairy farms in the U.S. marks a potential new phase in this epizootic. This strain of the virus has adapted to cattle and is transmitted through milk; several human cases have already been diagnosed. To date, human-to-human transmission of the virus has not occurred. Nevertheless, the question of a strain emerging that is better adapted to human-to-human transmission is being raised at a time when the U.S. appears to be paying less attention to its public and animal health situations. As Peacock *et al.* [19] have clearly demonstrated, the number of annual transmissions of influenza viruses, particularly certain strains of H5N1, to mammals has steadily increased since the 1990s. This further increases the risk that one of these strains will adapt to humans.

## Climate and deforestation

### Hendra Virus

The Hendra virus, a paramyxovirus belonging to the same genus as the Nipah virus, infects horses and humans, causing potentially fatal pneumonia in horses. First discovered in Australia in the 1990s, the virus was recently the subject of a 25-year study aimed at understanding its ecology and epidemiology and the factors explaining its sporadic emergence. While the number of human cases has remained low, the case fatality rate justifies expanding our knowledge of the associated risks. A study by Eby *et al*. [4] links the emergence of the virus to climatic and environmental factors that are increasingly correlated with human-induced parameters. These factors include deforestation following the arrival of Europeans on Australia’s east coast, ongoing forest fragmentation, and flowering patterns of local tree species, particularly those that bloom in winter, which are influenced by Southern Oscillation (ENSO, El Niño) variations. Based on these factors, Becker *et al.*’s study [2] proposes criteria for predicting years at risk for the virus’s emergence. The natural reservoir of the Hendra virus is the gray-headed flying fox (*Pteropus poliocephalus*), a large, fruit-eating Australian bat. Declines in natural forests and shortages of food resources (e.g., flowers and fruits) during certain winters and years force the bats to travel greater distances, including into orchards planted by fruit growers. This brings them closer to populated areas. Their immune systems are less effective at controlling viral replication when they are under physiological stress from increased searching for food. The animals can then more easily shed the paramyxovirus. Humans become infected through contact with horses that are exposed in their paddocks when these are planted with fruit trees that attract flying foxes. Horses likely become infected by consuming plant debris left behind by flying foxes, such as pieces of fruit contaminated by their saliva or fodder soiled by their droppings. Interestingly, despite tens of thousands of years of human habitation in Australia, present-day Aboriginal communities do not appear to be familiar with the virus, including those among whom hunting and consuming flying foxes was possible and practiced [14]. The virus appears to have existed well before the arrival of Europeans; however, recent environmental changes over the past two centuries may explain its emergence. These changes include the degradation of the reservoir’s habitat, indirect climate impacts *via* El Niño, the planting of orchards near inhabited areas, and the introduction of a domestic placental mammal, the horse, capable of transmitting the virus from the wild animal reservoir to humans. Currently, the risk of human exposure to the virus is higher in agricultural areas than in the least disturbed forests [4].

### Severe Acute Respiratory Syndrome (SARS-1 and SARS-2)

The lessons to be learned from the two SARS pandemics (SARS-1 in 2002–2003 and SARS-2, or SARS-CoV-2, since late 2019) have not all been documented yet [18]. The history and associated epidemiological data are certainly complex. However, the outbreaks originated in China in both cases, and international investigations and research had not materialized by the end of 2025. Both viruses are coronaviruses likely carried by bats. However, neither of the two viruses has yet been identified in bats, and the question of how they were transmitted to humans remains unresolved. In 2002–2003, a small Asian carnivore of the viverrid family, the masked palm civet (*Paguma larvata*), which had recently been raised in captivity in southern China, may have served as the link between bats and humans. For SARS-2, or COVD-19, there is still no answer to this question. The tradition of “wet markets,” where numerous animals of various species are kept in close quarters under poor sanitary conditions, was highlighted during that time. At the time, Chinese authorities announced that these high-risk practices would end. Yet, in 2019, when the first cases of the disease emerged, these practices could well have been still in place. Notably, no images circulated in late 2019 or early 2020 illustrating the reality of the implicated Wuhan market before the onset of the outbreak. Only images of the empty, cleaned-up market were shown.

In both cases, the viruses are of animal origin. However, after adapting to humans, they triggered two pandemics that spread without returning to the animal reservoir. Hypotheses explaining the origin of the SARS-CoV-2 pandemic combine work in southern Chinese mines where horseshoe bats (*Rhinolophus* spp.) roosted, fur farms housing animals such as raccoon dogs (*Nyctereutes procyonides*), and a laboratory leak or accident. These hypotheses are to be considered within the human, social, environmental, and economic context, which is undergoing rapid transformation in the country.

In the case of the SARS-CoV-2 virus, humans infected a wide range of domestic mammals (e.g., dogs and cats), farmed fur-animals (minks), pets (e.g., hamsters), and even wild animals (e.g., white-tailed deer in the U.S.) [17].

### Zoonoses transmitted by rodents

For certain existing zoonotic diseases, the risk is more direct and better understood, and predictions are based on more tangible evidence. This is the case for bacterial and viral diseases whose causative agents are carried by various species of rodents, whether wild or commensal. There are several possible epidemiological patterns with these species (more than 2,500 are known to date). For example, changes in rainfall patterns between the dry and rainy seasons in West Africa could lead to an increase in Lassa fever (*Mammarenavirus*, *Arenaviridae*) incidence in Nigeria^8^. Local authorities anticipate this. The responsible rodents are multi-mammary rats (*Mastomys* spp.), which are commensal. A recent study suggests that the current invasion of certain West African regions by black rats (*Rattus rattus*)—an introduced Asian species that competes with multimammate rats ecologically but is a poor reservoir for the virus—may reduce the human risk of Lassa fever [5].

Hantaviruses carried by European red voles (*Clethrionomys* spp.) could take advantage of the current expansion of forested areas and increased outdoor activity. Rodents that readily enter water could spread leptospirosis and amoebas (*Giardia* spp.) as they come into greater proximity with swimmers around the last remaining bodies of water accessible during periods of drought. Rather than the emergence of new risks, we need to be able to predict and anticipate the evolution of current risks (which ones and in what ways) [6]. July 15, 2022 press release from the National Academy of Medicine lists potential dangers^9^.

### Vector-borne zoonoses, insects, ticks

Vector-borne zoonoses are among the potential dangers. The scope of vector-borne diseases is vast. Until the end of the 20^th^ century, these diseases primarily affected countries in the intertropical zone and its peripheries. The transition to the 21^st^ century clearly signals that the area of influence of these disease-causing agents is expanding. The Mediterranean region and nearby countries with temperate climates are now affected.

One well-studied example is leishmaniasis along the shores of the Mediterranean [12] and in South America. Although the epidemiological data varies by country, the same categories of factors are present. Different species of sandfly vectors feed on different hosts. Although wild reservoirs still exist, the regular involvement of domestic dogs, which are always close to humans, illustrates the complex role of our species. In central Spain, *Leishmania infantum* leishmaniasis is spreading northward with the help of domestic dogs and populations of lagomorphs, such as the Iberian hare (*Lepus granatensis*) and the European rabbit (*Oryctolagus cuniculus*), which are sustained by hunting activities [3]. In southern France, the transformation of rural villages into residential areas surrounding major urban centers has benefited the vectors. Pet dogs are becoming increasingly prevalent [12]. In South America, a study focused on the “Amazonian deforestation arc” in the Brazilian state of Mato Grosso [13]. The list of potential vectors and reservoirs for local *Leishmania* species is long, but two important species stand out: the nine-banded armadillo (*Dasypus novemcinctus*) and the domestic dog. In this case, humans are indeed bringing the parasites and vectors into contact with themselves. The armadillo adapts to changes and survives in altered habitats, while the dog follows humans. These are well-known diseases, but their persistence and spread locally involve a combination of various factors, some of which are entirely anthropogenic. Furthermore, clearing natural areas that are uninhabited or sparsely populated can lead to disease emergence involving a previously unknown pathogen, though this is not the most common scenario.

In addition to monitoring the geographic spread of vector-borne diseases, it is crucial to anticipate potential outbreaks by establishing and enhancing surveillance networks for the vectors themselves. Medical and veterinary entomology has been neglected—or even ignored—for too long and must be brought back to the forefront. The *Aedes albopictus* case is a prime example.

The species is currently invading Europe dramatically, yet the expertise and tools needed to document and analyze this invasion must be present. In 2014, the species was present only along the Mediterranean coast of France. It has since spread to all 13 regions of mainland France. It is absent only from a few departments in the northwest and northeast^10^. The more than 750 locally acquired cases of chikungunya recorded in the summer of 2025 represent an all-time high for the country. France’s overseas territories, which are not discussed here, are living situations that mainland France is now gradually coming to resemble.

Expertise is needed to monitor and identify not only mosquitoes, but also culicoides, sandflies, black flies, and reduviids. Mites must not be overlooked. Tick-borne diseases are also evolving [11]. The recent identification and likely establishment of the *Hyalomma marginatum* tick in southern France is a cause for concern. This tick is a known reservoir and vector of Crimean-Congo hemorrhagic fever [26]. Collaborative work among physicians, epidemiologists, veterinarians, entomologists, ecologists, climatologists, elected officials, and citizens is necessary.

## Conclusion

Addressing the issue of possible links between environmental changes and the emergence of zoonoses raises numerous methodological questions. The list of examples presented here primarily illustrates the diversity of scenarios and the frequent interplay of several explanatory factors, including numerous anthropogenic parameters. Other approaches exist, such as the structured method proposed by Rupasinghe *et al.* [24]. Their final model combines 13 zoonotic pathogens with eight possible transmission routes: five vector-borne routes, plus foodborne, waterborne, and airborne routes. This serves as a reminder of two important yet distinct concepts: hazard and risk. The agents of canine rabies, bubonic plague, and hemorrhagic fever with renal syndrome are all hazards. However, the resulting risk—the probability of being bitten by a rabid dog, a black rat flea carrying *Yersinia pestis*, or inhaling an aerosol containing the Puumala virus—often depends on behaviors that expose certain humans to these hazards. The path from hazard to illness can take many routes. Good information and effective prevention make it possible to control and manage risks without necessarily seeking to eliminate the danger, or often, its reservoir or ecosystem. Finally, awareness is needed at all levels, from individuals to government officials.

## Acknowledgments

I would like to express my sincere gratitude to the organizers of this wonderful conference for their invitation, warm welcome, and availability. The event featured high-quality presentations and valuable interactions. I would also like to thank my colleagues at the SFMTSI for their support and constructive feedback.

## Funding

None

## Conflicts of interest

The author declares no conflicts of interest.

## Footnotes

1 https://www.ecologie.gouv.fr/actualites/publication-du-6e-rapport-synthese-du-giec

2 https://www.ipbes.net/global-assessment

3 https://www.thelancet.com/journals/langlo/article/PIIS2214-109X(25)00518-2/fulltext

4 GEO-7 https://www.unep.org/news-and-stories/press-release/ un-report-investing-planetary-health-would-deliver-higher-gdp-fewer

5 https://www.assistancescolaire.com/eleve/5e/histoire/reviser-une-notion/5hdm03

6 https://rabiesalliance.org/

7 https://www.woah.org/fr/article/stepping-up-dog-population-management-to-achieve-rabies-elimination/

8 ProMED, 29 décembre 2024

9 https://www.academie-medecine.fr/entre-le-bien-etre-du-rat-degout-et-la-sante-publique-faut-il-choisir/

10 https://sante.gouv.fr
